# Acquired immunity and Alzheimer's disease

**DOI:** 10.7555/JBR.36.20220083

**Published:** 2022-07-28

**Authors:** Weixi Feng, Yanli Zhang, Peng Sun, Ming Xiao

**Affiliations:** 1 Jiangsu Key Laboratory of Neurodegeneration, Nanjing Medical University, Nanjing, Jiangsu 211166, China; 2 Institute of Neuroscience, State Key Laboratory of Neuroscience, CAS Center for Excellence in Brain Science and Intelligence Technology, Shanghai Research Center for Brain Science and Brain-Inspired Intelligence, Chinese Academy of Sciences, Shanghai 200031, China; 3 Brain Institute, Nanjing Brain Hospital, Nanjing Medical University, Nanjing, Jiangsu 210029, China

**Keywords:** Alzheimer's disease, acquired immunity, lymphocytes, brain lymphatic system, immune cell therapy

## Abstract

Alzheimer's disease (AD) is an age-related neurodegenerative disease characterized by progressive cognitive defects. The role of the central immune system dominated by microglia in the progression of AD has been extensively investigated. However, little is known about the peripheral immune system in AD pathogenesis. Recently, with the discovery of the meningeal lymphatic vessels and glymphatic system, the roles of the acquired immunity in the maintenance of central homeostasis and neurodegenerative diseases have attracted an increasing attention. The T cells not only regulate the function of neurons, astrocytes, microglia, oligodendrocytes and brain microvascular endothelial cells, but also participate in the clearance of β-amyloid (Aβ) plaques. Apart from producing antibodies to bind Aβ peptides, the B cells affect Aβ-related cascades *via* a variety of antibody-independent mechanisms. This review systemically summarizes the recent progress in understanding pathophysiological roles of the T cells and B cells in AD.

## Introduction

Alzheimer's disease (AD) is an age-related neurodegenerative disease characterized by progressive cognitive defects. Epidemiological surveys have shown that about 15% to 20% of 65-year-olds have mild cognitive impairment (MCI), of whom about 32% will develop AD within five years^[1]^. Pathological changes of AD mainly consist of β-amyloid (Aβ) plaque deposition, neurofibrillary tangles caused by tau hyperphosphorylation, loss of neurons and synapses, and neuroinflammation in memory-related brain areas, but the exact mechanisms leading to these pathological changes have not been fully elucidated yet^[2]^. Current studies have shown that an imbalance between the production and degradation of brain parenchymal metabolites may be one of the pathogenesis of AD^[3]^. Notably, the balance of metabolism is influenced by mitophagy. A causative role of defective mitophagy in AD is supported by the evidence that the turning-up of mitophagy inhibits AD progression^[4]^. Disruption of the brain homeostasis leads to the accumulation of metabolic products such as Aβ and tau proteins within brain parenchyma, resulting in neuronal and synapse loss and cognitive impairments. In addition to direct neurotoxicity, abnormal accumulation of metabolites can cause neuroinflammation, further exacerbating neurodegenerative progression^[5–6]^. Especially, impaired mitophagy, including neuronal mitophagy and microglial mitophagy, activates the inflammation pathway *via* activation of NACHT, LRR and PYD domains-containing protein 3 (NLRP3) and reduction of phagocytotic activities^[7–8]^. Therefore, neuroinflammation is one of the key events in the development of AD^[9–11]^.

Indeed, previous studies have suggested that neuroinflammation occurs in the middle and late stages of AD^[12–13]^. However, recent studies have shown that immune alterations may trigger the occurrence of AD^[14–18]^. Genome-wide association studies (GWAS) have shown that many genes, such as *TREM2*, *CR1*, *CD33*, *SPI1* (*PU.1*), *EPHA1*, and *MS4A4A*/*MS4A6A*, increase AD risk and are related to the immune system^[19–22]^. These genes have also been shown to be involved in AD pathogenesis. For example, TREM-2 can promote activation, survival and phagocytosis of microglia in the AD process^[23–24]^, and in microglia, hyper-activation of CD33 inhibits microglial phagocytosis of Aβ plaques^[19]^. In addition to the elimination of metabolites, microglia can directly engulf dendritic spines, and regulate synaptic number and plasticity, which is abnormally enhanced in the process of AD^[25–27]^. Moreover, PU.1 is reported to be associated with microglial state transition and Aβ clearance^[28]^ and its mutation regulates several AD-associated genes expressed in human myeloid cells and may be in the causal path to AD^[29]^. These results suggest that microglia-related neuroinflammation can promote the occurrence or development of AD.

The brain was previously considered an immune-exempt organ. However, with the discovery of the cerebral lymphatic system^[30–38]^, there is some increasing evidence that meningeal lymphatic vessel-mediated acquired immunity participates in physiological functions and pathological changes in the central nervous system (CNS). This article will systematically review the role of the acquired immunity in the occurrence and development of AD, and prospect the corresponding treatment strategies that target the acquired immune cells.

## Epidemiological evidence of the acquired immunity in Alzheimer's disease

The acquired immune system is mainly composed of both T cell and B cell immunities. Epidemiological evidence has indicated that the total number of T cells and B cells in the peripheral blood of AD patients is significantly reduced, compared with those in the healthy controls, while other acquired immune cells, such as natural killer (NK) cells, do not show significant changes^[39–42]^. In addition, proinflammatory cytokines, such as tumor necrosis factor-α (TNF-α) secreted by lymphocytes, are significantly increased in AD patients^[43–44]^. For different T cell subgroups, although the changes of CD8^+^ cytotoxic T cells in the process of AD remain elusive, many studies have found an increase in peripheral blood CD4^+^ helper T cell subsets in prodromal AD, and this increase was gradually reversed in the late stage of AD^[43]^. Another study also found that CD27 and CD28 double positive CD4^+^ T cells were decreased in AD patients, while senescence associated killer cell lectin-like receptor subfamily G member 1 (KLRG1) positive and CD57 positive CD4^+^ T cells were increased^[41,45]^. These results suggest that AD-related pathology may stimulate the hyper-responsiveness of CD4^+^ T cells; however, under continuous stimulation, which in turn may result in their progressive depletion.

The changes of B cells in the peripheral blood of AD patients have also been reported^[40,42]^. The AD-related toxic proteins such as Aβ can stimulate B cells to secrete specific antibodies, and CD19^+^ total B cells are reduced in the peripheral blood of AD patients, which may reduce the secretion of specific antibodies against AD pathology, thereby exacerbating the development of AD. IgD^+^CD27^−^ naive B cells are significantly reduced when AD progressing to the middle and late stages, while IgD^−^CD27^−^ memory B cells are significantly increased^[42,46]^. This change may damage the protective effect of B cells on AD pathology. The single nucleotide polymorphisms of AD risk genes obtained by GWAS analysis also suggest an involvement of B cells in the development of AD^[47–48]^. Moreover, the proportion of CD4^+^ T cells or CD19^+^ B cells is positively correlated with the MMSE score, a marker of cognitive function, in AD patients^[42–43]^. The above results suggest that the acquired immunity based on T cells and B cells participates in AD-related pathological processes.

## Acquired immune cell infiltration into the central nervous system

In the physiological state, there are a large number of lymphocytes in the dura mater, subarachnoid space and pia mater, thus making the physiological state of brain parenchyma monitorable at any time^[34,49]^. There are about 15 000 T cells in the cerebrospinal fluid (CSF) of normal people, of which about 70% are CD4^+^ T cells^[50–52]^. The pathology of brain vascular offers the opportunity for the entrance of T cells into the brain. For example, the expression of the blood-brain barrier (BBB)-related genes, such as *ZO1* (zonula occludens-1, ZO-1) and occludin, are decreased due to cerebral amyloid angiopathy (CAA) in AD^[53–54]^, which makes T cells easy to penetrate into the brain. In addition, peripheral T cells derived from AD patients overexpress chemokine receptor CXCR2, which contributes to their recruitment to the pathological sites^[55]^. Importantly, it is reported that extravascular CD8^+^ T cells are present in the perivascular space of blood vessels with CAA in the hippocampi of AD brains^[49]^. These results confirm the possibility of T cell entrance into the brain in AD. Interestingly, T cells can pass through the subpial space or subarachnoid blood vessels into the subarachnoid space^[56]^, or directly enter the subarachnoid space through the pia mater in experimental autoimmune encephalomyelitis^[57]^. But whether T cells can infiltrate into the CNS in this way in AD still needs further investigation. In addition, the recently discovered glymphatic system demonstrates that para-vascular space is the place for the transport of CSF into and out of the brain^[30]^. However, whether these pathways could allow for the infiltration of lymphocytes still needs more studies.

A number of studies have shown that the BBB damage caused by AD pathology results in peripheral lymphocytes infiltrating into the subpial space, hippocampus and cortex of AD patients and animal models^[49,58–60]^. Under normal conditions, brain microvascular endothelial cells are wrapped by the vascular basement membrane, pericytes, and end feet of astrocytes, which work together to maintain the BBB integrity. Studies have reported that the elevation of TNF-α in the perivascular space or subarachnoid space can cause the recruited myeloid cells to up-regulate matrix metalloproteinase 2 (MMP-2) and MMP-9, selectively lysing the proteoglycans on the end feet of astrocytes. This will promote the opening of the BBB, and facilitate immune cells to migrate into the brain parenchyma^[61–62]^. Connexins anchor each other between microvascular endothelial cells, which can restrict soluble substances and cells to pass through the BBB^[63]^. However, in neurodegenerative diseases, such as AD, Aβ is transported from the brain into the blood *via* vascular endothelium, which could lead to its accumulation and the appearance of CAA. These Aβ deposits near the endothelial cells could decrease the expression of ZO-1 directly, or induce inflammation and impair the permeability of BBB subsequently^[54]^. Such changes will promote the immune cells in the subarachnoid space or peritubular space to enter the brain parenchyma^[64–65]^.

The entry of lymphocytes from the peripheral blood into the subarachnoid space is mainly regulated by the choroid plexus or meningeal blood vessels. The choroid plexus is a specific structure located in the ventricles of the CNS. The blood vessels on the pia mater or ependyma repeatedly branch to form plexiforms in some parts, together with the pia mater and ependymal epithelial cells, protruding into the brain ventricle to form the choroid plexus. The epithelial cells that make up the choroid plexus with high permeability, can filter blood in the vessels, and form and secrete CSF^[66]^. The choroid plexus constitutes the brain-cerebrospinal fluid barrier and restricts penetration of immune cells under normal circumstances^[67]^. In AD patients, the choroid plexuses are larger than those in controls and the severity of cognitive impairment is associated with larger choroid plexus volume^[68]^. In AD models, it is reported that Aβ in the CSF induces nitric oxide generation and increases reactive oxygen species of choroid plexus^[69]^. Importantly, intracerebroventricular injection of Aβ results in the injury of choroid plexus and upregulation of chemokines^[70]^, which may contribute to the recruitment of lymphocytes into the CSF.

**Figure 1:**
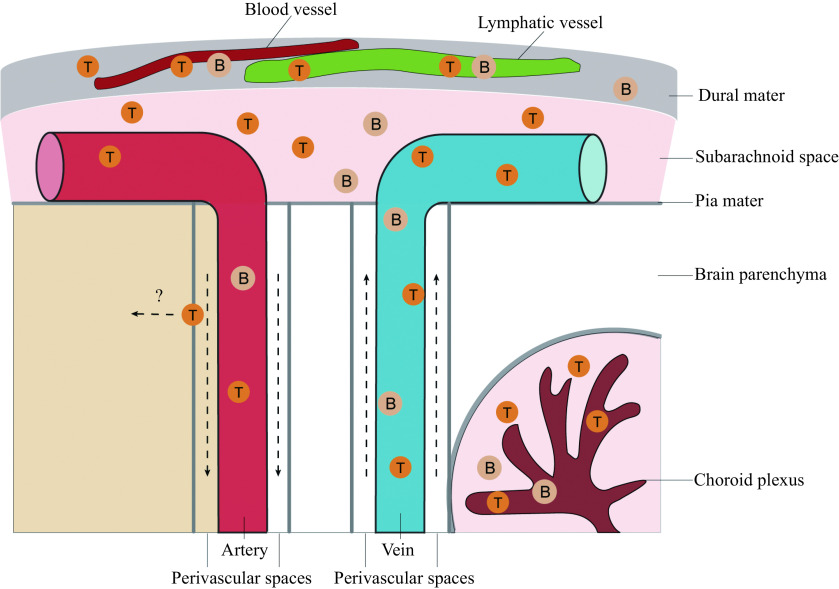
Acquired immune cells in the central nervous system.

Meningeal blood vessels are another pathway for lymphocytes to enter the subarachnoid space^[71]^. Studies have reported that although the activation and detachment of T cells from the meninges are necessary for the cells to enter the parenchyma, but the pathway of these T cells to the parenchyma is still unclear^[72]^. However, under neuroinflammatory conditions, the chemokine gradient induced by meningeal immune cells may be one of the mechanisms that cause lymphocytes to pass through the BBB into the brain parenchyma^[50]^. The above-mentioned pieces of evidence together suggest that peripherally acquired immune cells can enter the CNS under physiological or pathological conditions to regulate brain function and microenvironment homeostasis (***Fig. 1***).

## Central antigen drainage

The CNS has been considered an immune-exempt organ due to the lack of traditional lymphatic vessels. One study showed that transplantation of allogeneic tissues into the cortex of MHC-incompatible recipient rats did not cause immune rejection, which suggests that the brain parenchyma lacks acquired immune surveillance^[73]^. The lack of draining lymphatic vessels is considered the main reason for immune exemption in the CNS^[74]^. However, a series of recent findings have amended/subverted the definition of the CNS immune privilege. Several studies have reported that there is a vessel-like structure expressing traditional lymphatic endothelial cell markers, such as lymphatic vessel endothelial hyaluronan receptor-1, prospero-related homeobox transcription factor 1, and vascular endothelial growth factor receptor 3 in the dura. These meningeal vessels are responsible for draining macromolecular substances from the brain parenchyma and immune cells within CSF^[31,34]^. Blocking lymphatic drainage of the meningeal lymphatic system *via* physical or chemical methods will aggravate massive accumulation of toxic proteins, such as Aβ, in the brain parenchyma and aggravate pathological progression in the AD mouse model^[35–36]^.

**Figure 2:**
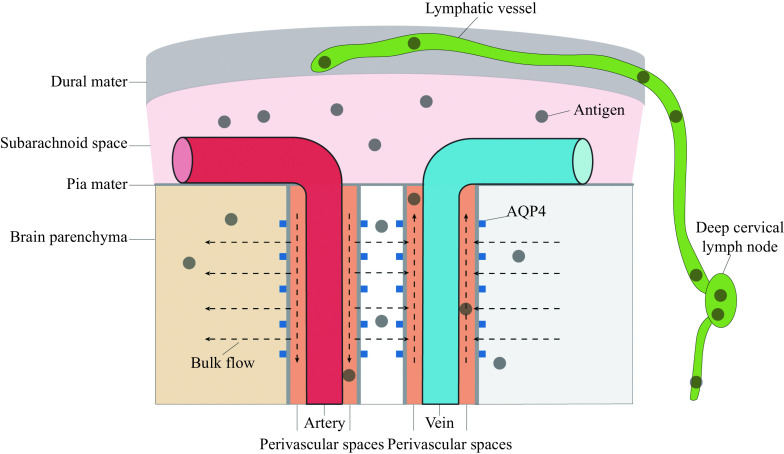
Central antigen drainage.

More interestingly, there is also a brain-wide fluid transport pathway, known as the glymphatic system, in the brain parenchyma^[30]^. The glymphatic system consists of three principal sequential anatomic segments: (i) CSF inflow along the perivascular spaces surrounding penetrating arteries; (ii) dispersion of CSF through the wider interstitium; and (iii) facilitated by aquaporin 4 on the vascular endfeet of astrocytes, efflux of interstitial fluid along the large-caliber draining veins to re-enter the CSF within the ventricular and cisternal compartments. Therefore, central soluble antigens and metabolites may enter the meningeal lymphatic vessels, and finally reach the cervical lymph nodes to activate the adaptive immune system. Studies have reported that damages in the meningeal lymphatic transport reduce the content of central antigens in the cervical lymph nodes and the activation of MOG-specific T cells^[75]^, which further proves that central antigens can enter the peripheral circulation through the glymphatic-meningeal lymphatic routes. However, the types of central antigens and the mechanism of regulating lymphatic drainage of antigens still need to be further studied (***Fig. 2***).

## An involvement of the acquired immune cells in Alzheimer's disease

### T cells and Alzheimer's disease

CNS inflammatory diseases, such as multiple sclerosis (MS), are usually accompanied by massive infiltration of brain parenchymal T cells. However, more and more studies have demonstrated the presence of T cells in the brain parenchyma of patients with neurodegenerative diseases such as AD and Parkinson's disease (PD)^[49,58]^. The initial study reported that T cells mainly surrounded the Aβ plaques^[76]^, but the subsequent studies have found that there are extensive T cells in the brain parenchyma of AD patients, especially the temporal cortex and hippocampus^[77]^.

#### The influence of T cells on Aβ pathology

Aβ can act as an antigen to activate lymphocytes in the course of AD. Epidemiological investigations have shown that compared with that in the middle-aged and young people aged 25–40, the activation of Aβ-specific T cells in the elderly and AD patients is significantly increased^[78]^. Animal experimental studies have revealed that the role of T cells in the Aβ pathology is dependent on their specific differentiation states. Studies have shown that T helper type 1 (Th1) cells can aggravate the deposition of brain parenchymal plaques and impair the learning and memory of the AD model mice. The investigators used Aβ to stimulate T cells to differentiate into Th1 types *in vitro*, and then transplanted the differentiated cells into six- to seven-month-old APP/PS1 mice through the vein, proving that the transplanted Th1 cells aggravate plaque deposition in the hippocampus and cortex by secreting interferon-gamma (IFN-γ)^[79]^. However, contrary to this conclusion, another research group found that after injection of Th1 cells into the lateral ventricle of 12- to -15-month-old APP/PS1 mice, the transplanted T cells can quickly enter the brain parenchyma through the action of MHC-II molecules and the adhesion molecule ICAM-1, and these transplanted T cells can persistently accumulate near the plaques for four weeks, and accelerate the clearance of the Aβ plaques by promoting glial cell phagocytosis, thereby reducing the load of the Aβ plaques^[80]^. These conflicting conclusions may be due to differences in the locations of cell injection or recipient mice. The short-term infiltration of Th1 cells from the ependymal location may promote local inflammation, enhance the phagocytic ability of glial cells and facilitate plaque clearance; however, the role of Th1 cells derived from peripheral blood other than CSF in the pathogenesis of AD still needs further research.

Compared with Th1 cells, the role of Aβ-specific Th2 cells in AD is basically clear. Many studies have shown that Th2 cells can reduce Aβ through an interleukin-4 (IL-4) dependent mechanism, thereby improving cognitive impairment. For example, the transplantation of Aβ-specific Th2 cells could significantly reduce Aβ plaque deposition in the brain parenchyma of 11-month-old APP/PS1 mice, and improve the cognitive function of the recipient mice^[81]^. Artificial induction of Th2 cell differentiation also could reduce Aβ in the brain parenchyma of Tg2576 mice and improve their cognitive ability^[82]^.

Studies have also reported the role of Aβ-specific Th17 cells in AD. The injection of Aβ_1–42_ in the hippocampus significantly increases the infiltration of Th17 cells into the parenchyma; however, this method of Aβ immunization usually leads to meningoencephalitis, which may cause a strong Th1 cell response^[83–84]^. The phenotype analysis of T cells in the circulating blood of AD patients have shown that CD25^+^ CCR6^+^ Th17 cells are significantly increased, and these phenotypes of Th17 cells have been reported to promote neuronal damage in MS and PD^[85]^. In addition, investigators have reported an obvious infiltration of Th17 cells in the hippocampus of an AD rodent model, and also reported that IL-17 and IL-22 secreted by Th17 cells aggravate the loss of neurons in the CA1 area of the hippocampus and the activation of glial cells^[86]^. Moreover, it is known that Fas ligand (FasL) is highly expressed in the hippocampal Th17 cells of AD model animals, and that neurons with similar high Fas expression may undergo apoptosis through the Fas-FasL pathway^[86]^. In summary, Aβ, an important pathological indicator of AD, can induce T cells to differentiate into different types of cells, such as Th1, Th2, and Th17, and different types of Th cells can participate in the regulation of AD-like pathological processes through different mechanisms.

#### The effect of T cells on neurons

Th1 and Th2 cells have been shown to produce a variety of neurotrophic factors to promote the development, function and survival of neurons. For example, T cells can enhance the expression of brain-derived neurotrophic factor under the stimulation of central antigens, thereby inducing the growth of axons and supporting the survival of neurons^[87]^. IL-2 derived from Th2 cells has been shown to reverse the damaged synaptic plasticity and enhance dendritic spine density in the brain parenchyma of APP/PS1 mice^[88]^. In addition, T regulatory cells (Tregs) can also inhibit CNS inflammation and maintain neuronal function through immunosuppressive mechanisms^[89]^.

T cells can also directly cause damage to the neurons or dendritic spines in a variety of ways. The neuronal JAK/STAT1 pathway is essential for the phagocytosis of synapses by microglia. A recent experiment has shown that CD8^+^ cytotoxic T cells infiltrate the brain parenchyma and activate the neuronal JAK/STAT1 pathway *via* secretion of IFN-γ, up-regulate the expression of CCL2, and promote the recruitment of microglia, subsequently causing synaptic phagocytosis and neuronal damage^[90]^. More importantly, there is also evidence of increased neurofilament-M (NF-M) in the CSF and serum of MCI patients and AD patients, which can predict the prognosis of AD patients in its early stage^[91–92]^. However, others have also found that in a mouse model of MS, autoreactive myelin-specific T cells can recognize neuron skeleton antigen NF-M, which may damage neurons directly^[93]^. Therefore, whether the increased NF-M will promote these differentiated T cells to damage neurons, and whether this T cell-mediated damage is one of the reasons for the clinical symptoms of AD remain to be further studied.

#### The effect of T cells on glial cells

In AD, it is known that Th cells can regulate the response of glial cells by directly contacting or releasing cytokines. Aβ_1-42_ stimulates differentiated Th1 cells to migrate into the brain parenchyma, induce local inflammation, and stimulate the activation of microglia. A mild activation of microglia increases their phagocytic ability, which helps microglia to surround and clear the Aβ plaques more effectively^[94]^. In addition, IFN-γ derived from Th cells can induce the expression of TREM-2 and signal-regulatory protein beta-1 in microglia and significantly increase the phagocytic ability of microglia^[95–96]^. Unlike Th1 cells, transforming growth factor beta (TGF-β) secreted by Treg cells has a variety of functions, including anti-inflammatory, regulating microglia phenotype conversion, *etc.*^[97]^. In addition, IL-4 and IL-23 secreted by Th cells can act directly as anti-inflammatory mediators, leading to a decrease in the levels of pro-inflammatory factors IL-1β, IL-6, TNF-α, and reactive oxygen species secreted by glial cells. Experiments have shown that an intracerebral injection of IL-4 and IL-13 secreted by Th cells can significantly up-regulate insulin-like growth factor 1 levels and reduce Aβ deposition^[98]^. In addition, T cells have also been reported to be involved in the phenotypic regulation of macrophages. For example, T cells can down-regulate the levels of macrophage inflammasomes NLRP1 and NLRP3 to reduce caspase-1-mediated release of the pro-inflammatory factor IL-1β^[99]^. However, whether these effects of T cells have the same effects on microglia still needs to be determined.

Similarly, T cells can regulate astrocyte physiological and pathological functions in AD. Glutamate derived from T cells can act on astrocytes to promote the neuroprotection of activated astrocytes^[100]^. There are also reports that astrocytes can affect survival and functions of T cells. Experiments have shown that when CD4^+^ T cells and astrocytes are co-cultured, T cells will differentiate in the direction of Th1 cells^[101]^, and experiments have also found that astrocytes can maintain the expression of Foxp3 in T cells through the IL2-STAT5 pathway, promoting survival of the Tregs^[102]^. Recent experiments have also shown that Tregs can affect the functions of oligodendrocytes in the EAE model. For example, the differentiation and myelination of oligodendrocytes in Tregs deficiency mice could be significantly impaired after EAE modeling^[103]^. Further studies have confirmed that Tregs can mediate the differentiation of oligodendrocyte precursor cells through cellular communication network factor 3^[103]^. Because AD is also accompanied by loss of myelin sheath, whether dysfunction of Tregs exists in the process of AD requires further investigations.

The activation of T cells requires the stimulation of antigen-presenting cells. There are reports that microglia can act as antigen-presenting cells to stimulate the differentiation of T cells and recruit lymphocytes to infiltrate^[104]^. IL-17 secreted by Th17 cells has also been found to promote microglia to secrete C-C motif chemokine ligand 2 (CCL2), CCL12, and C-X-C motif chemokine ligand 2 (CXCL2), and increase its recruitment function for lymphocytes^[105]^. In addition, IFN-γ derived from Th1 cells can stimulate the expression of MHC class II molecules, CD40, and CD86 in microglia^[94,104]^, induce the secretion of chemokines CXCL10, CCL2, and CCL5, and in turn, enhance the activation and recruitment of T cells^[106]^. In the AD model, the Aβ plaques can stimulate the microglia around the plaques to up-regulate the expression of CD40^[107]^. The elevated CD40 on the surface of microglia can bind to CD40 ligand (CD40L) on the surface of T cells to promote their activation^[108]^. Although astrocytes are not immune cells, there is also evidence that astrocytes can up-regulate the expression of MHC class II molecules after being stimulated by IFN-γ^[109]^. It has been reported that astrocytes can act as antigen-presenting cells to activate myelin-specific T cells* in vitro*, and this effect can be canceled by CD80 or CD86 blockers^[110]^. In summary, in addition to typical dendritic cells and other antigen-presenting cells, microglia and astrocytes may act as antigen-presenting cells in AD pathology, stimulating the proliferation and differentiation of T cells after infiltrating the brain parenchyma. On the other hand, different types of T cells that infiltrate the brain parenchyma can also regulate the protective or harmful effects of glial cells, thereby affecting the progression of AD.

#### The effect of T cells on vascular endothelial cells

In addition to undergoing phagocytosis and being cleared by glial cells, Aβ can also be transported by vascular endothelial cells to enter the circulatory system and be cleared in the periphery. A variety of receptors on vascular endothelial cells, such as low density lipoprotein receptor 1, ATP-binding cassette transporter A1 (ABCA1), receptor of advanced glycation endproducts (RAGE), can mediate the mutual transport of Aβ between the brain and the circulatory system. When the homeostasis of this transport system is damaged, cerebral amyloid angiopathy (CAA) will occur. A previous study has reported that there is an accumulation of Th17 cells in the brain parenchyma of AD mice, and the secreted cytokine IL-17 is found to accumulate around Aβ plaques; furthermore, overexpression of IL-17 can significantly promote the expression of ABCA1 in vascular endothelium, accelerate the transport of Aβ from the brain parenchyma to the circulatory system, and reduce CAA lesions in the process of AD^[111]^. On the contrary, vascular endothelial cells can also affect the functions of T cells in a variety of ways. Endothelial cells can stimulate the differentiation of T cells, and TGF-β1 can reduce the differentiation ability of Th1 cells induced by endothelial cells and reduce the secretion of IFN-γ of Th1 cells^[112]^.

In addition, under AD pathology, vascular endothelial cells can also regulate the infiltration of T cells across the BBB. For example, T cells in the peripheral blood of AD patients overexpress CCL5, inducing cerebral vascular endothelial cells to express the corresponding receptor CCR5, and mediating T cells through the BBB *via* CCL5/CCR5 pathway^[113]^. And Aβ can also interact with vascular endothelial RAGE and induce the up-regulation of CCR5^[113]^. CD4^+^ T cells have also been found to directly up-regulate the expression of ICAM-1 within vascular endothelial cells and promote their adhesion and infiltration in the blood vessel wall^[80]^. Blocking the interaction of T cell VLA-4 and vascular endothelial cell VCAM-1 by antibodies can effectively reduce the infiltration of CD4^+^ and CD8^+^ T cells into the brain parenchyma of 3×Tg-AD mice, and reduce the deposition of Aβ and the phosphorylation of Tau^[114]^.

**Figure 3:**
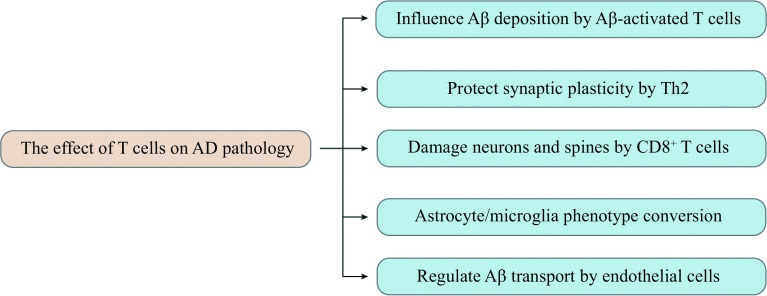
The effect of T cells on Alzheimer's disease pathology.

In summary, T cells can induce the expression of vascular endothelial cell chemokines and adhesion molecules, promote their passing across the BBB, or promote the expression of Aβ transport-related molecules, and affect the accumulation of toxic products in the brain parenchyma. Moreover, vascular endothelial cells can also regulate phenotypic differentiation of central infiltrating T cells in a cytokine manner (***Fig. 3***).

### B cells and Alzheimer's disease

Because B cells have long been considered specialized antibody-secreting cells, the role of B cells in AD has not received much attention. However, epidemiological investigations have shown that certain human leukocyte antigen (HLA) alleles, such as HLA-DRB1*15:01, are high-risk factors for AD^[115]^. Studies have found that the level of B cell subsets in the peripheral blood of some AD patients is reduced^[42]^, which may be related to genetic factors of these cells. These results suggest that B cells may play a regulatory role in the occurrence and development of AD.

#### Antibody-dependent B cell function

Antibodies secreted by B cells can penetrate the BBB in small quantities and passively diffuse into the healthy brain parenchyma^[116]^. Compared with healthy controls, the levels of Aβ_1-42_ antibodies in the serum or CSF of AD patients are significantly reduced^[117–118]^. This difference may be related to the binding of Aβ antibody to Aβ_1-42_ in AD patients, because the combination of antibody and Aβ_1-42_ will result in a significant decrease in free antibody levels and soluble Aβ_1-42_ levels^[119]^. This phenomenon can provide a theoretical basis for the reduction of soluble Aβ_1-42_ levels in the CSF of patients with an advanced AD, and also support the potential role of anti-Aβ antibodies in the treatment of AD. However, the presence of antibodies in the brain parenchyma has a 'double-edged sword' effect. On the one hand, immunoglobulin is a potential non-specific immunosuppressant^[120–121]^. It can transmit immunosuppressive signals by binding to FcγRIIB, a low affinity receptor that recognizes the Fc portion of IgG, on the surface of target cells and stimulate the production of immunosuppressive factors^[122]^. An animal study has shown that non-amyloid-reactive IgG can promote the ability of microglia to bind and engulf Aβ, while 5×FAD mice lacking B cells and T cells have more Aβ plaque deposition^[123]^. This also provides a theoretical basis for the protective effect of AD transgenic mice injected with non-specific IgG to eliminate Aβ. On the other hand, antibodies that enter the brain parenchyma also have a damaging effect. In the process of AD, Fc receptors on the surface of microglia and peritubular macrophages are up-regulated, and peripheral-derived antibodies can bind to these receptors to induce inflammatory reactions^[124]^. For example, antibodies against synaptic NMDA receptors can aggravate the cognitive and psychiatric symptoms of patients with progressive cognitive dysfunction^[125]^.

#### The function of antibody-independent B cells

In addition to secreting antibodies, B cells have other functions, such as regulating inflammation through the secretion of cytokines, and stimulating the differentiation of T cells as antigen-presenting cells. B cells can secrete IFN-γ and TNF-α, inhibit regulatory immune cells, and induce macrophages to differentiate toward pro-inflammatory phenotype^[126]^. B cells can also present their antigens to T cells^[127]^ and induce the differentiation of CD4^+^ or CD8^+^ T cells^[128–129]^. However, whether these functions of B cells are affected during aging is still unclear. Studies have found that the increase in the age-related senescent B cells in old mice can induce the differentiation of Th1 and Th17 cells^[130]^, and the number of IgD^+^CD27^+^ B cells with senescent phenotypes increased with an AD-like process^[42]^. A recent study has demonstrated a decreased number of B cells in the blood of AD patients, or B cell depletion in the early stage *via* intraperitoneal injection of anti-CD19/B220 antibodies into APP/PS1 transgenic mice markedly accelerates cognitive dysfunction and Aβ burden^[131]^. However, another study reported that by neutralizing B cells with B220/CD20 antibodies, 5×FAD mice showed some reduced IgG in the hippocampus of 35- to 47-week-old 5×FAD mice and an increased number of anti-inflammatory microglia, leading to a decrease in the Aβ plaques and subsequently improved cognitive dysfunctions^[132]^. So, the role of B cells in AD pathology may be complex and needs more investigations. In addition, it has been shown that B cells that are positive for MHC class Ⅱ molecules can induce the differentiation of myelin-specific T cells, and the removal of B cells inhibits demyelination and axon loss caused by activated T cells^[133–134]^. Similarly, B cells have also been shown to promote the occurrence of stroke-related cognitive dysfunction^[135–136]^. However, the contribution of B cells to AD process and the underlying mechanism remain to be elucidated.

**Figure 4:**
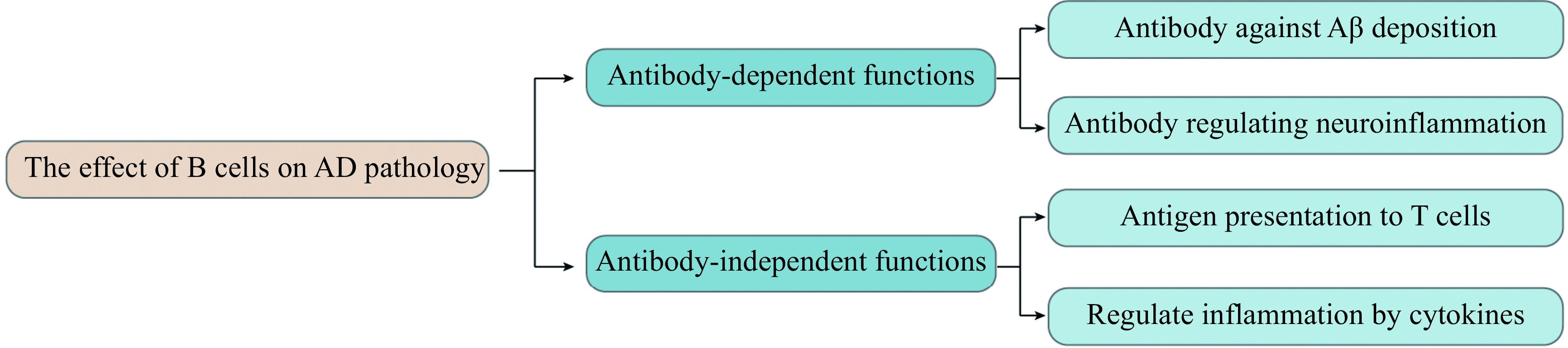
The effect of B cells on Alzheimer's disease pathology.

B cells can also play a protective role through an immunosuppressive function. Regulatory B cells (Bregs) are a type of cells that can secrete anti-inflammatory factors. Bregs can be divided into different types, such as CD1d^high^CD5^+^ B10 cells, CD43^−^CD21^high^CD23^low^CD2^high^IgD^+^IgM^+^CD93^int^CD1d^high^CD19^+^ transitional-2 marginal zone B-cell precursor cells (T2-MZP Bregs), CD5^−^B220^low^CD11b^+^CD23^−^CD24^+^CD62L^+^IgM^+^CD1d^high^ B1b cells, Tim-1^+^CD1d^high^CD5^+^ Bregs, IL-33^+^ Bregs, and IL-35^+^ Bregs^[137]^. Most of the immunosuppressive ability of these Bregs can be enhanced by the activation of Toll-like receptors (TLRs)^[138]^, and Aβ can also act as a ligand to bind to TLR4^[139]^, but whether it can induce the proliferation and differentiation of Bregs has not been reported so far. TGFβ secreted by Bregs is also an important immunosuppressive factor. TGFβ can form a monomer or form a complex with immunoglobulin to suppress host immunity. It is reported that Bregs can induce the production of myeloid-derived suppressive cells through the TGF-β signaling pathway^[140]^. In addition, Bregs have been reported to induce and maintain the expression of FoxP3 through IL-10, and promote the proliferation and differentiation of FoxP3^+^ Tregs^[141]^. Bregs have also been reported to inhibit Th1 and Th17 cells through IL-35, and promote the formation of Tregs^[142–143]^. In addition, IL-35 and other anti-inflammatory factors with immunosuppressive function can act on the regulatory cells themselves to promote the secretion of IL-10 with anti-inflammatory function^[144]^. However, the role of this immunosuppressive function of B cells in AD is rarely reported. A recent article has reported that Tau can activate B1a cells, and that IL-10 secreted by B1a cells can effectively reduce inflammation^[145]^. However, whether B cells can affect the pathological process of AD through other mechanisms still needs more investigations (***Fig. 4***).

## Concluding remarks and perspectives

The functions of natural immune cells, especially microglia, in AD have been extensively studied. However, the role of the acquired immune system in AD remains elusive. The innate immune cells are rapidly activated in response to an acute infection, but the subsequently activated lymphocyte-based acquired immune system may be crucial in resistance to the long-term stimuli. Based on this, alterations in the acquired immune system with aging may play a major role in those aging-related disorders, including AD.

The effects of the AD-related pathology on peripheral acquired immunity are still rarely reported. With the discovery of the cerebral lymphatic system, physiological or pathological antigens from the brain can be drained from the meningeal lymphatic vessels into the cervical lymph nodes, subsequently inducing the systemic immune response. In the process of AD, whether the antigenic components in the local lymph nodes will be changed, how toxic macromolecules damage the brain lymphatic transport and antigen presentation functions, and whether the lymphocyte activation and physiological function will be correspondingly affected, are worthy of further study.

In clinical trials, the use of synthetic Aβ_1-42_ vaccine significantly reduces the brain parenchymal Aβ deposition^[146]^, but the infiltration of T lymphocytes produced by immunity causes the subjects to develop severe aseptic meningitis^[147]^. At present, this active immunization method is undergoing improvements, such as immunogenic modification, to reduce the inflammatory response and the production of cytotoxic Aβ-specific T cells. Programmed death protein-1 (PD-1) and programmed death ligand-1 (PD-L1) exist on the surface of immune cells and non-immune cells to regulate immune tolerance and immunity^[148]^. Studies have reported the effects of anti-PD-1 or anti-PD-L1 antibodies in 5×FAD mouse models. It has been reported that inhibition of the PD-L1/PD-1 pathway causes peripheral IFN-γ-dependent systemic immune response and promotes the recruitment of peripheral blood mononuclear-macrophages into the brain parenchyma. Macrophages with high expression of scavenger receptors can rapidly clear misfolded toxic proteins including Aβ and apoptotic cells, and inhibit the excessive inflammation of the brain parenchyma through the secretion of anti-inflammatory factors. They also can secrete a variety of growth factors to ameliorate the brain pathology and improve the cognitive functions of the AD model mice^[149]^. Therefore, unlike the AD antibody-targeted therapy, the PD-L1/PD-1 targeted therapy can simultaneously ameliorate a variety of pathological changes in the brain parenchyma^[150]^, which may help reverse the AD process. However, the effectiveness and safety of the PD-L1/PD-1 therapy in clinical application still need more detailed investigations.

In summary, considerable progress has been achieved in exploring the role of lymphocyte-based acquired immunity in the pathogenesis of AD. The regulatory role of the brain lymphatic system in the interaction between CNS and the acquired immune system remains to be investigated. Targeting the acquired immunity may open a new avenue to delay or even prevent pathological progress of AD and improve cognitive impairment.
